# Functional Dynamics of Arginine Mono- and Di-Methylation

**DOI:** 10.3390/cells14110796

**Published:** 2025-05-29

**Authors:** Xi’ang Wang, Bin Zhu, Robert Winn, Shanfa Lu, Hengbin Wang

**Affiliations:** 1 Department of Internal Medicine, Division of Hematology, Oncology and Palliative Care, Massey Comprehensive Cancer Center, School of Medicine, Virginia Commonwealth University, Richmond, VI 23298, USA; 2 State Key Laboratory for Quality Ensurance and Sustainable Use of Dao-di Herbs, Institute of Medicinal Plant Development, Chinese Academy of Medical Sciences & Peking Union Medical College, Beijing 100193, China; 3 Department of Internal Medicine, Division of Pulmonary Disease and Critical Care, Massey Comprehensive Cancer Center, School of Medicine, Virginia Commonwealth University, Richmond, VI 23298, USA

**Keywords:** methylation, arginine methylation, di-methylation, mono-methylation, PRMTs

## Abstract

Arginine methylation is a crucial post-translational modification (PTM) that plays a significant role in various biological processes. It occurs in two primary forms: mono-methylation (MMA) and di-methylation (DMA), with the latter further classified into symmetric (SDMA) and asymmetric methylation (ADMA). This review examines the functional implications of these methylation states, current detection methodologies, proteomics-based analytical approaches, and the different impacts of these methylations on protein function. Finally, the role of protein arginine methyltransferases (PRMTs) and their substrate specificity in shaping the arginine methylome are discussed.

## 1. Introduction

Post-translational modification (PTM) plays a pivotal role in modulating protein functions across numerous biological processes. Through covalent conjugation of small chemical groups or proteins to specific amino acid residues, PTMs alter protein activity, localization, and interactions. Among the most common PTMs are methylation, ubiquitination, acetylation, phosphorylation, and hydroxylation [1]. The discovery of protein methylation dates back to 1959 [2], and with the advancement of detection technologies, an expanding number of methylation sites have been identified, predominantly on arginine and lysine residues. Arginine methylation exists in three distinct forms: monomethylarginine (MMA), asymmetric dimethylarginine (ADMA), and symmetric dimethylarginine (SDMA) [3]. These modifications are made possible by the guanidinium group in the arginine side chain, allowing for unique structural and functional outcomes depending on the methylation pattern [3]. The diversity of these methylation states underpins the involvement of arginine methylation in a wide range of cellular and physiological processes.

The biological significance of arginine methylation, particularly its involvement in disease, has been discussed in several recent reviews [4,5,6,7]. While di-methylation (DMA) plays prominent roles in transcriptional regulation, RNA splicing, and the DNA damage response, MMA appears to be more involved in signal transduction and early-stage splicing events [5,7]. This functional divergence implies that MMA and DMA are not merely sequential modifications but also distinct regulatory elements. Dysregulation of either type can contribute to disease pathogenesis: for instance, ADMA is implicated in muscle atrophy and kidney diseases, whereas SDMA is more associated with prostate cancer and glioblastoma. Both MMA and DMA have been shown to contribute to breast cancer development [6].

In this review, we provide a comprehensive overview of the methodologies used to detect arginine methylation and analyze its type-specific functions. We explore the distinct biological roles of MMA and DMA, highlighting their differential impacts on protein structure and function. Furthermore, we delve into the catalytic mechanisms of protein arginine methyltransferases (PRMTs) responsible for MMA and DMA generation. By dissecting the interplay between enzyme specificity, structural biology, and cellular context, we aim to shed light on the complex regulatory network governed by arginine methylation and its implications in disease development.

## 2. Detection and Analysis of Arginine Methylation

The methylation of arginine residue results in only minor changes in molecular mass, with 14 Da for MMA and 28 Da for DMA [8]. These small differences are insufficient to produce noticeable shifts in protein migration on traditional detection methods such as SDS-PAGE. As the resolution has increased, cryo-electron microscopy (Cryo-EM) has become a useful tool to detect chemical modifications with high reliability. The RNA modifications in human and *E. coli*’s ribosomes are detected clearly [9,10]. Moreover, the highest resolution of Cryo-EM is up to 1.25 Å, which means every protein atom can be visualized precisely, including the methylation sites [11]. However, this method needs high purity and a large number of samples to obtain a high resolution over 2.5 Å. It is unsuitable for large-scale methylation detection. Consequently, mass spectrometry (MS) has become the gold standard for detecting and characterizing methylated arginine residues, owing to its high sensitivity and precision.

In addition to MS-based approaches, the development of methylation-specific antibodies has significantly advanced the detection and enrichment of methylated proteins. These antibodies, designed to specifically recognize mono- and di-methylated arginine residues, have enabled large-scale proteomic analyses through applications such as immunoprecipitation (IP), Western blotting, and immunofluorescence. When combined with heavy methyl-stable isotope labeling by amino acids in cell culture (hm-SILAC), these antibodies offer improved sensitivity and specificity, facilitating a more comprehensive mapping of the arginine methylation site [12,13,14,15].

### 2.1. Advances in Proteomics Data Analysis

**Figure 1:**
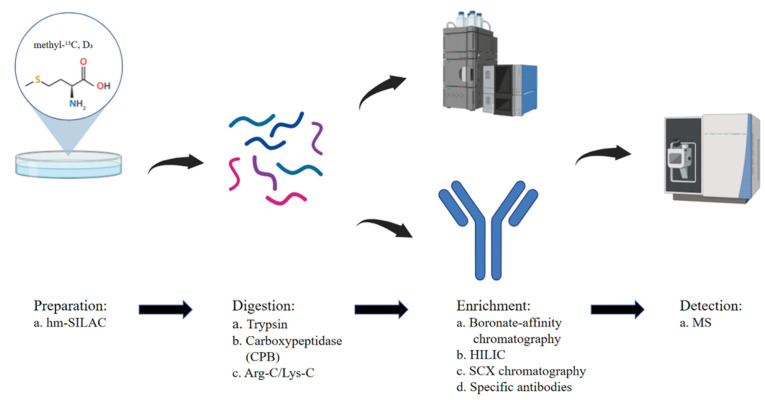
Workflow for the detection of arginine methylation using hm-SILAC and mass spectrometry. Cells are metabolically labeled with heavy methyl-labeled methionine (hm-SILAC: methyl-^13^C, D_3_), enabling differentiation of methylation events. Proteins are then digested using proteases such as trypsin, carboxypeptidase B (CPB), or Arg-C/Lys-C. Following digestion, methylated peptides are enriched through multiple strategies including boronate-affinity chromatography, hydrophilic interaction liquid chromatography (HILIC), strong cation exchange (SCX) chromatography, or immunoprecipitation with methylation-specific antibodies. Enriched peptides are subsequently analyzed by mass spectrometry (MS) to identify and quantify arginine methylation sites with high sensitivity and specificity.

The first large-scale proteomic analysis of arginine methylation was conducted in 2004, using methylation-specific antibodies, stable isotope labeling by amino acids in cell culture (SILAC), and liquid chromatography–tandem MS. This study identified 59 methylation sites, making a pivotal advancement in the field [16]. By 2012, the integration of hydrophilic interaction liquid chromatography (HILIC) with ILAC-based mass spectrometry enabled the detection of hundreds of methylation sites, significantly expanding our knowledge of the arginine methylome [17]. Further advancements, such as strong cation exchange (SCX) chromatography and immune-affinity purification (IAP), have played critical roles in refining methylation detection [12,13,14,15,18,19,20,21]. A comparative analysis of high-pH SCX and IAP demonstrated that these approaches are complementary and exhibit high quantitative reproducibility, underscoring the necessity of combining both methods for a comprehensive global assessment of protein methylation [22] (Figure 1).

Despite these advancements, several challenges remain, including histidine interference, the loss of methylated peptides, and other limitations that impact detection accuracy. To address these issues, imidazole carbonylation and hydrazide resin treatments have been used to deplete histidine-containing peptides, while electron-transfer dissociation (ETD)-based proteomic workflows help minimize neutral losses [23,24]. Additionally, steric effect-based chemical enrichment methods (SECEM) enable the specific detection of demethylated arginine and differentiation between ADMA and SDMA [25]. Hydrophobic labeling techniques have been employed to extend the retention time during reversed-phase chromatography before MS analysis, improving detection sensitivity [26]. Furthermore, methyl-neutron-coding (mNeuCode) tagging, a novel labeling approach, enhances the specificity of methylated arginine detection [27]. Most recently, a chemoenzymatic strategy for global enrichment and the identification of all three types of arginine methylation has been developed, leading to the discovery of 1006 arginine methylation events, including 645 demethylated sites and 361 monomethylated sites [28]. These ongoing methodological improvements continue to refine our understanding of arginine methylation site distribution and its functional significance.

### 2.2. Quantification and Distribution of Methylation Sites

**Figure 2:**
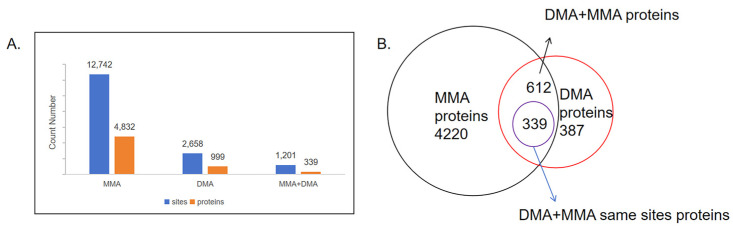
Overview of proteins and sites identified with mono- and dimethylarginine modifications. (**A**) Bar graph showing the total number of arginine methylation sites and corresponding proteins identified for MMA, DMA, and those carrying both MMA and DMA modifications. Blue bars represent the number of modified sites, while orange bars represent the number of modified proteins. (**B**) Venn diagram illustrates the overlap between proteins bearing MMA and DMA modifications. Of the 999 DMA-modified proteins, 612 also contain MMA modifications, with 339 proteins exhibiting both MMA and DMA at the same methylation sites.

We collected proteins with methylated arginine from the 15 published proteomic data (see Data Availability Statement). Following duplicate removal and classification, 12,742 MMA sites across 4832 proteins and 2658 DMA sites across 999 proteins are summarized (Figure 2A, Supplemental Table S1). MMA sites are approximately three times more abundant than DMA sites, reinforcing the notion that DMA formation is typically derived from MMA [18,29]. Venn diagram analysis further demonstrates that over 60% of DMA-containing proteins also exhibit MMA, supporting the hierarchical nature of this modification process. However, 387 proteins contain only DMA sites (Figure 2B), indicating that certain MMA intermediates may be short-lived, raising questions about the distinct physiological significance of MMA and its role in cellular processes.

Interestingly, 1201 arginine residues across 339 proteins have been identified with both MMA and DMA, highlighting the dynamic nature of arginine methylation. However, the mechanisms governing this transition and its functional consequences remain unclear. One possible explanation is that MMA serves as an intermediate stage in the progression toward DMA modification [30]. On average, proteins with only MMA sites contain 2.6 methylation sites, while those with only DMA sites have 2.2 sites. However, proteins that harbor both MMA and DMA at the same sites exhibit a significantly higher average of 3.5 methylation sites, suggesting a selective enrichment of dynamic methylation in specific proteins. Additionally, 273 proteins contain both MMA and DMA sites, though these modifications occur at distinct arginine residues rather than the same sites, suggesting site-specific regulation of arginine methylation.

## 3. Functional Implications of Arginine Methylation

### 3.1. Impact on Protein Properties

**Figure 3:**
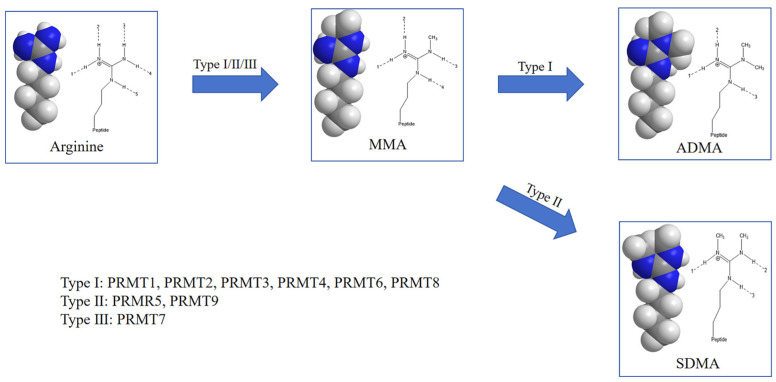
Types of protein arginine methylation and corresponding PRMT classifications. Schematic representation of the stepwise methylation of arginine residues by protein arginine methyltransferases (PRMTs). All PRMT types initially catalyze the formation of MMA. Type I PRMTs (PRMT1, PRMT2, PRMT3, PRMT4, PRMT6, and PRMT8) further catalyze the formation of ADMA, while Type II PRMTs (PRMT5 and PRMT9) convert MMA to SDMA. Type III PRMT (PRMT7) catalyzes only the formation of MMA. The molecular structures shown depict the side-chain modifications at each methylation state.

The guanidinium group of arginine carries a positive charge and can form up to five hydrogen bonds, as well as π-stacking interactions (Figure 3). While methylation does not alter the charge state of the guanidinium head but rather modifies steric conformation, charge distribution, and hydrophobicity of the molecule [7,31]. Compared to MMA, both ADMA and SDMA exert stronger effects on the physical properties of arginine. The overall volume increase in the Arginine head group is 17% for MMA, 33% for ADMA, and 34% for SDMA (Figure 3). Likewise, methylation significantly influences hydrophobicity, as reflected in the logP value, which shifts from −0.34 (unmodified arginine) to 0.18 (MMA), 0.56 (ADMA), and 0.70 (SDMA), demonstrating that DMA increases hydrophobicity approximately threefold compared to MMA. Furthermore, ADMA and SDMA exhibit more diffuse charge localization, which may further affect molecular interactions [31].

Interestingly, despite these changes, arginine methylation does not strongly impact the pKa value of the guanidinium group. Instead, steric effects dominate over electronic effects, leading to significant conformational alterations [8,32]. As a result, arginine methylation modulates protein function by altering the physical properties of the modified residues. For instance, ADMA can block interactions between arginine and aromatic residues, resulting in a more extended conformation and a decrease in liquid–liquid phase separation (LLPS) [33]. Additionally, methylation can disrupt protein dimerization, such as in malate dehydrogenase 1 (MDH1), where methylation at the dimeric interface prevents inter-subunit hydrogen bonding [34]. Furthermore, arginine methylation influences protein–protein and protein–nucleic acid interactions [4].

In enzymatic contexts, methylation at catalytically active arginine residues can directly affect enzymatic activity. For example, MMA at R236 of phosphoglycerate dehydrogenase (PHGDH) enhances catalytic activity, promoting serine biosynthesis, even though PHGDH mRNA levels and protein content decrease upon knockdown [35]. Similarly, ADMA at R112 on lactate dehydrogenase A (LDHA) positively impacts catalytic efficiency [36].

Overall, arginine methylation primarily alters protein function through steric effects, yet the functional distinctions between MMA and DMA remain unclear. Further investigation is necessary to understand the precise roles of different methylation states in regulating protein structure and function.

### 3.2. Diverse Functions of MMA and DMA

#### 3.2.1. Overview

**Figure 4:**
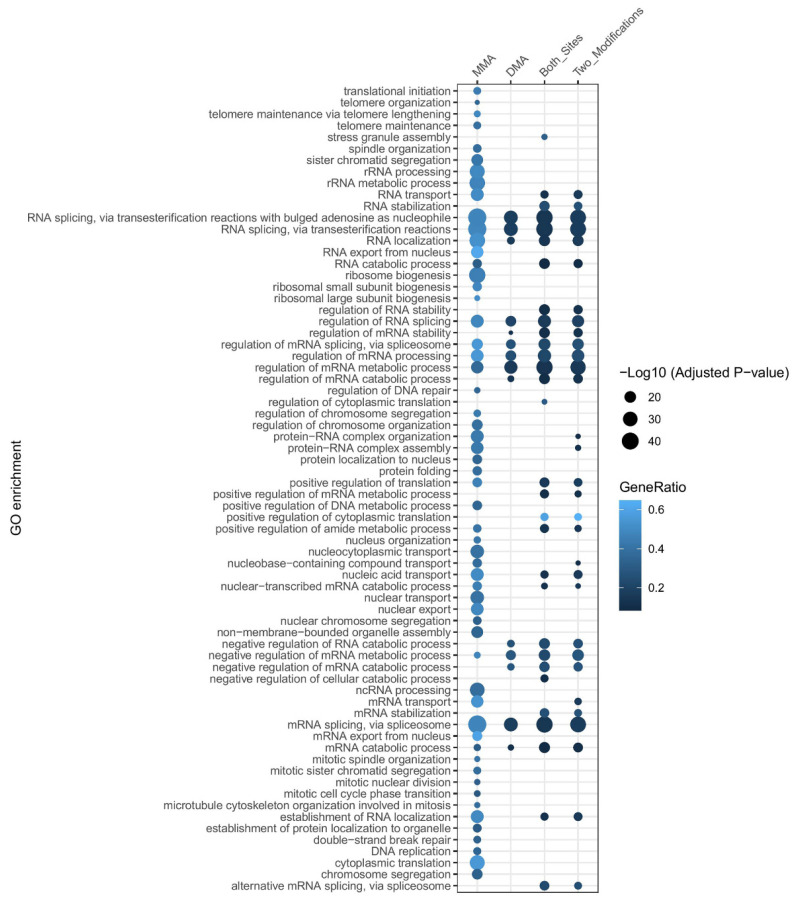
Gene Ontology (GO) enrichment analysis of proteins with MMA, DMA, or both modifications. Dot plot illustrating enriched biological processes (GO terms) associated with proteins harboring MMA, DMA, or both methylation types. Dot size represents the significance level of enrichment (−log_10_ adjusted *p*-value), while the color intensity indicates the GeneRatio (the proportion of genes associated with each GO term). Biological processes related to RNA metabolism, splicing, and transport are prominently enriched across all methylation types. MMA, MMA-involved proteins; DMA, DMA-involved proteins; both sites, the proteins contain at least one site on which both MMA and DMA occur; two modifications, the proteins containing both MMA and DMA, regardless of whether these two modifications happened on the same site.

To elucidate the biological processes influenced by MMA and DMA, we performed Gene Ontology (GO) analysis on proteins containing these modifications. Additionally, we analyzed proteins that harbor both MMA and DMA sites or sites modified by both mono- and di-methylation. All these proteins for GO analysis are from the collection mentioned above (see Section 2.2 *Quantification and Distribution of Methylation Sites*). Biological pathway enrichment was performed using the compareCluster function in R, and *p*-values were adjusted by the Benjamini–Hochberg method. The entire set of annotated human genes was used as a background reference to calculate gene enrichment ratios. Biological pathways with a *p*-value below 1 × 10^−10^ were selectively displayed (Figure 4, Supplementary Tables S2 and S3).

The results indicate that most enriched biological processes, regardless of methylation type, are associated with nucleic acid-related functions, which can be attributed to the physicochemical properties of arginine. The pathways enriched by proteins containing both MMA and DMA modifications are highly similar to those enriched by proteins in which both modifications occur at the same site. Otherwise, for pathways enriched by proteins containing DMA modifications, more than 90% overlap with pathways enriched by proteins containing MMA modifications. (Figure 4). This raises the question of whether MMA serves solely as an intermediate state or has distinct functional roles.

Further analysis revealed that MMA-associated proteins are enriched in ribosome biogenesis, RNA localization, cytoplasmic translation, and nucleocytoplasmic transport, suggesting a strong influence of MMA on RNA transport and gene translation (Figure 4). In contrast, DMA-modified proteins are primarily enriched in the regulation of mRNA metabolic processes, mRNA processing, and negative regulation of mRNA metabolism, indicating that DMA plays a key role in gene expression regulation. For proteins where both MMA and DMA modifications occur at the same site, GO analysis highlights the unique enrichment in RNA stabilization, regulation of RNA stability, and mRNA stabilization. This suggests that the transition between MMA and DMA is crucial for RNA stability regulation (Figure 4).

#### 3.2.2. Tissue-Specific and Disease-Related Variability in Arginine Methylation

Most identified methylated arginine peptides originate from common experimental cell lines such as HeLa, HEK293T, or Jurkat T cells. To better understand the role of arginine methylation in specific biological processes, proteomics studies have been conducted in various tissues and organs. In colorectal cancer (CRC) patient tissue pools, 455 MMA and 314 asymmetric dimethylarginine (ADMA) sites were identified, with enrichment in mRNA splicing and mRNA processing pathways [37]. These findings indicate that arginine methylation profiles differ between in vivo patient-derived samples and in vitro cell line models, such as the HCT116 CRC cell line [37]. Similarly, ADMA profiles also differ between breast cancer cell lines and patient-derived xenograft (PDX) tumors, regardless of estrogen receptor (ER) status (ER^+^ or ER^−^). Interestingly, most identified ADMA-modified proteins in breast cancer models are also involved in mRNA processing, reinforcing the idea that ADMA primarily regulates transcriptional processes [38]. Additionally, arginine methylation patterns exhibit organ specificity, even though some modification sites are conserved across at least four different organs [39]. In conclusion, arginine methylation patterns vary across tissues and diseases, leading to changes in protein function and cellular development. These findings emphasize the need for further research into the biological implications of tissue- and disease-specific arginine methylation modifications.

## 4. PRMTs and Their Role in Arginine Methylation

Proteins catalyzed by arginine methylation are protein arginine methyltransferases (PRMTs). The nine PRMTs are categorized into three types based on their enzymatic activity. Type I PRMTs include PRMT1, PRMT2, PRMT3, PRMT4/CARM1, PRMT6, and PRMT8, which catalyze ADMA. Type II PRMTs include PRMT5 and PRMT9, which catalyze ADMA. Type III PRMTs include PRMT7, which exclusively catalyzes MMA (Figure 3) [6]. Although Type I and Type II PRMTs are primarily associated with DMA, some studies have shown that PRMT1 and PRMT5 can also mediate MMA, as observed in substrates like TDP-43 and DUSP14 [40,41]. This section further explores the functional differences among PRMTs, their roles in MMA vs. DMA, and the structural mechanisms underlying these modifications.

### 4.1. Functional Specificity of PRMTs

Among the nine PRMTs, PRMT1 and PRMT5 are the primary enzymes responsible for DMA, with PRMT1 accounting for over 50% of normal cellular ADMA levels [8,42]. Due to their significant role in regulating methylation, the methylation sites controlled by these two PRMTs have attracted extensive research interest.

#### 4.1.1. PRMT1 and PRMT5 in Arginine Methylation Regulation

In HEK293T and HeLa cells, PRMT1 knockdown and inhibitor treatment resulted in a substantial decrease in ADMA levels, with a two-fold reduction in the intensity of 158 DMA sites affecting 49 proteins [22,28]. The fractional occupancy of methylated arginine sites, a measure of methylation extent at specific residues, dropped from 26% to 10% following PRMT1 and PRMT5 knockdown, underscoring their strong enzymatic control over arginine methylation stoichiometry [18]. Besides regulating ADMA levels, PRMT1 deficiency also influences MMA and SDMA levels. Similarly, PRMT5 inhibition alters both DMA and MMA modifications [23]. A total of 114 MMA sites exhibited significant upregulation or downregulation, particularly in proteins involved in mRNA metabolic processes [22]. Some MMA sites disappeared following PRMT1 knockdown, indicating that PRMT1 can also catalyze MMA [41].

#### 4.1.2. Functional Redundancy and PRMT Interplay

**Figure 5:**
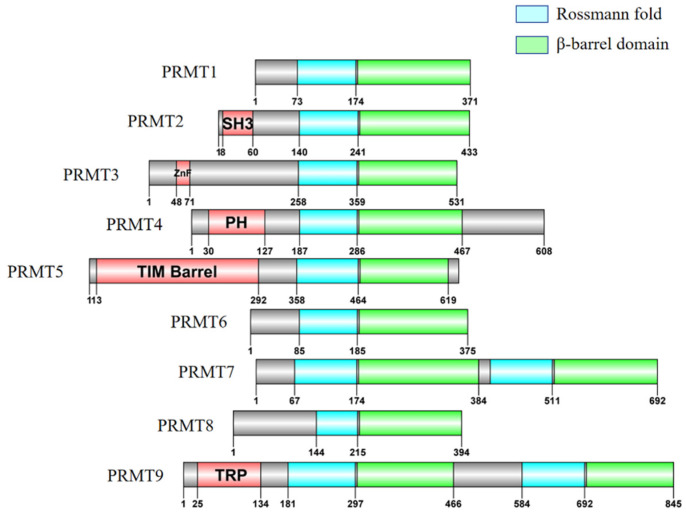
Domain architecture of human PRMT family members. Schematic representation of the domain structures of protein arginine methyltransferases (PRMTs) 1–9. All PRMTs contain a conserved Rossmann fold (blue) and β-barrel domain (green), which together form the catalytic core responsible for methyltransferase activity. Additional functional domains (red) are present in specific PRMTs: SH3 domain in PRMT2, zinc finger (ZnF) in PRMT3, pleckstrin homology (PH) domain in PRMT4, TIM barrel in PRMT5, and TRP domain in PRMT9. These variable domains may contribute to substrate specificity, protein–protein interactions, and subcellular localization. Numbers indicate amino acid positions. The other regions of each PRMT are shown in gray.

The increase in MMA levels upon PRMT1 inhibition could be attributed to compensatory effects by other PRMTs, such as PRMT5 and PRMT7. Comparative methylome analyses of PRMT4, PRMT5, and PRMT7 knockdown cell lines identified 62 commonly regulated proteins, suggesting functional overlap among these PRMTs [43]. It is also possible that some ADMA sites catalyzed by PRMT1 undergo methylation by other Type I PRMTs following PRMT1 loss. Although mass spectrometry may not detect significant changes in methylation levels, over 70% (214 out of 280) of peptides in peptide array experiments were methylated by at least one of PRMT1, PRMT4, or PRMT6, with one-third of these peptides being shared substrates. Notably, PRMT1 and PRMT6 share over 80% of their substrates [38], likely due to their structural similarity, as both contain only a catalytic core region without additional domains (Figure 5). Additionally, PRMT6 exhibits broader substrate specificity than PRMT1 and PRMT4 due to its larger binding pocket [44].

#### 4.1.3. Site-Specific Methylation and PRMT Crosstalk

PRMT1 knockdown led to notable changes in both ADMA and SDMA levels, with an overall increase in SDMA in PRMT1-deficient cells. Interestingly, the hnRNPA1 R206 methylation site underwent a shift modification from ADMA to SDMA following PRMT1 knockdown [22]. This site is also regulated by PRMT7 (MMA), PRMT4 (ADMA), and PRMT5 (SDMA), but PRMT4 failed to methylate it in vitro, indicating a level of substrate specificity among PRMTs [43,45]. Similarly, the RGG/RG motif of SERBP1 contains both ADMA and SDMA, and mutating these residues to lysine resulted in a reduction in both modifications [23]. These findings highlight the highly dynamic nature of arginine methylation, where multiple PRMTs can target the same residue to ensure proper protein function and cellular homeostasis. However, several key questions remain: which type of methylation is the “native” or functionally dominant modification? What signals drive the switch between MMA and DMA? How does methylation state alteration impact protein function and cellular processes?

#### 4.1.4. Functional Specialization of PRMTs

Gene Ontology (GO) analysis of PRMT-regulated substrates indicates that PRMT1, PRMT4, PRMT5, PRMT6, and PRMT7 are primarily enriched in nucleic acid-associated processes, such as RNA processing, splicing, and binding, transcription and translation regulation, and RNA localization and stability control [18,22,23,28,38,43,45,46]. Additionally, PRMT-specific methylation patterns highlight their distinct functional specializations. For example, PRMT1 is enriched in nitrogen compound transport, RNA localization, and protein localization; PRMT4 can selectively methylate proteins involved in p53-mediated signal transduction; PRMT5 primarily methylates proteins associated with cell cycle regulation; and PRMT7 uniquely methylates proteins related to utero embryonic development [22,43]. These findings underscore the functional complexity and redundancy of PRMTs, with substrate sharing and context-dependent regulation playing essential roles in arginine methylation dynamics. The proteins whose arginine methylation levels are influenced by their respective PRMTs are also summarized, but whether the methylated sites in these proteins are directly targeted by the given PRMT remains to be further validated (Supplementary Table S4). Understanding how PRMTs interact with and compensate for one another will be key to unraveling the full biological significance of this modification.

### 4.2. Structural Basis of PRMT Activity

The distinct functions of protein arginine methyltransferases (PRMTs) in the methylome are largely attributed to their ability to catalyze MMA and DMA, which in turn is influenced by their structural differences. The first reported PRMT structure was that of PRMT3 in 2000 [47], and since then, extensive structural investigations have shed light on the molecular basis of PRMT activity.

A typical PRMT core structure consists of two primary domains: the N-terminal Rossmann fold (AdoMet-binding domain) and the C-terminal β-barrel domain. The α-helical dimerization arm, which extends from the C-terminal β-barrel domain, interacts with the N-terminal Rossmann fold and is critical for PRMT dimerization and enzymatic activity (Figure 5). Dimerization is essential for PRMT function, except for PRMT7, which forms a pseudo-dimer [48,49]. For instance, PRMT5 forms a hetero-octameric complex with MEP50 (methylosome protein 50) to ensure substrate specificity [50]. In contrast, PRMT7 contains two PRMT core structures in tandem, where only the N-terminal PRMT core is catalytically active, while the C-terminal core lacks AdoMet-binding ability [51,52] (Figure 5). Interestingly, oligomerization differences among Type I/II PRMTs (PRMT1, PRMT5) and Type III PRMTs (PRMT7) may correlate with their ability to catalyze mono- vs. di-methylation.

A recent study demonstrated that PRMT1 exists as monomers, dimers, and tetramers, with dimeric PRMT1 being catalytically active and its efficiency varying between dimeric and tetrameric states depending on the substrate [53]. Mutations in the dimerization arm (W197L, Y202N, and M206V) hinder dimer formation and AdoMet binding, significantly reducing PRMT1 activity [54]. However, *Trypanosoma brucei* PRMT7 (TbPRMT7), which lacks the tandem C-terminal module, still forms a homodimer and exclusively generates MMA [55]. This evidence rules out oligomerization as the sole determinant of PRMT7’s mono-methylation activity, even though it may influence PRMT function.

#### 4.2.1. Catalytic Motifs and Substrate Recognition

**Figure 6:**
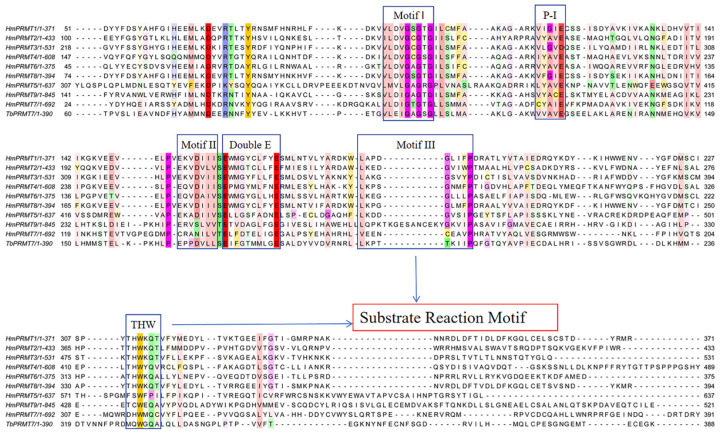
Multiple sequence alignment of PRMT family members highlighting conserved catalytic and substrate-binding motifs. Sequence alignment of Homo sapiens PRMTs 1–9 and *Trypanosoma brucei* PRMT7 (TbPRMT7) reveals conserved motifs essential for methyltransferase activity. Key functional motifs are boxed: Motif I, Motif II, Motif III, the double E loop, and the THW loop, which are critical for S-adenosylmethionine (SAM) binding and catalysis. The conserved P-I (post-I) region and the substrate recognition motif are also indicated. Colored residues denote sequence conservation, with strong conservation shown in magenta and variability in other colors. This alignment underscores the evolutionary conservation of the PRMT catalytic core and substrate-binding interface across species and PRMT types.

Sequence alignment of PRMTs reveals six conserved motifs in the catalytic core region, with all except the THW motif being located in the N-terminal Rossmann fold. These motifs include Motif I (VLD/VGxGxG) which forms the AdoMet-binding site with three strictly conserved glycine residues; Post-motif I (P-I) (V/I-X-G/A–X-D/E), which facilitates hydrogen bonding with AdoMet via a conserved glutamic/aspartic acid residue; Motif II (E/K/VDII), which stabilizes Motif I by forming a β-sheet interaction; Double-E motif (SExMGxxLxxExM), which positions arginine for methylation, with two glutamic acid residues facilitating substrate binding; Motif III (LK/xxGxxxP), which enhances enzyme stability by interacting with Motif II; and the THW loop, which plays a critical role in substrate recognition and stabilization of the active site (Figure 6) [48]. The two glutamic acid residues in the double-E motif, along with the histidine in the THW loop, are particularly important for substrate binding and catalysis. The negatively charged glutamic acids form a salt bridge with the positively charged Nη1 atom of arginine, anchoring the substrate in the reaction dock toward AdoMet [47].

#### 4.2.2. Free-Energy Barriers and Methylation Specificity

The specificity of mono- vs. di-methylation is dictated by the free-energy barrier of methyl group transfer, which is influenced by the spatial distance(r) and angular orientation (θ) between arginine’s nitrogen atoms (Nη1 and Nη2) and AdoMet’s S−CH3 group (Table 1). In PRMT3, the free-energy barrier for MMA formation is lower at Nη2 (3.6Å, 37.4°) than Nη1 (4.9Å, 54.6°), favoring initial methylation at Nη2. The second methylation event also favors Nη2 due to its optimal spatial parameters (3.2Å, 11.7°), resulting in a free-energy barrier of 18.4 kcal/mol [56]. PRMT5 follows a similar pattern, sequentially methylating Nη1 and Nη2 to form SDMA, with free-energy barriers of 20.4 kcal/mol and 20.1 kcal/mol, respectively [57]. PRMT7, however, exhibits a much higher free-energy barrier (32.7 kcal/mol) for MMA formation at Nη2, and the barrier for the second methylation is even higher (40.3 kcal/mol for ADMA and 44.3 kcal/mol for SDMA). This suggests that PRMT7 fundamentally lacks catalytic efficiency to generate DMA [58] (Table 1). Interestingly, PRMT7 mutants (E181D and E181D/Q329A) acquire the ability to catalyze DMA, with free-energy barriers reduced to 36.14 kcal/mol (ADMA) and 32.62 kcal/mol (SDMA), indicating that structural modifications can alter PRMT specificity (Table 1) [59,60,61]. Further free-energy simulations reveal that these mutations optimize substrate positioning, reducing r and θ values, thereby facilitating the second methylation event.

#### 4.2.3. Functional Implications of PRMT Mono- vs. Di-Methylation

Proteomic data indicate that MMA sites are more prevalent than DMA sites, suggesting that Type I and Type II PRMTs also catalyze mono-methylation extensively. Notably, the free-energy barrier for MMA formation in PRMT5 and PRMT3 is as low as that in PRMT7, implying that PRMT5 and PRMT3 can readily generate MMA [56,57,58]. Given the structural similarity between PRMT3 and PRMT1, it is reasonable to infer that PRMT1 also has a low free-energy barrier for MMA. However, the free-energy barrier for the second methyl transfer in PRMT1, PRMT3, and PRMT5 is significantly lower than for MMA formation, suggesting that these enzymes preferentially catalyze DMA [56,57,58]. This raises an important question: Why do Type I and Type II PRMTs sometimes stop at MMA rather than progressing to DMA? One possibility is that MMA at certain sites is not merely an intermediate step toward DMA but may have independent biological functions.

Additionally, the relative abundance of MMA sites in proteomic datasets could be due to their dynamic regulation. It is possible that although Type I/II PRMTs catalyze a greater number of MMA sites, DMA occurs more consistently at a subset of sites, making them easier to detect. Conversely, for PRMT7, the high free-energy barrier for DMA formation (>40 kcal/mol) suggests that its function is restricted to MMA generation, and it does not naturally support subsequent methylation events [58]. The interplay between PRMT structure, substrate binding, and free-energy dynamics plays a critical role in defining MMA vs. DMA. While Type I and Type II PRMTs favor DMA, they also catalyze a substantial number of MMA events. The reasons behind their selectivity in stopping at MMA vs. proceeding to DMA remain unclear, warranting further investigation.

## 5. Regulation and Dynamics of Arginine Methylation

The positioning of the substrate within the reaction dock of PRMTs is critical for determining the specificity and outcome of arginine methylation. Both key catalytic residues of PRMTs and the structural characteristics of substrate peptides play essential roles in shaping product specificity. The distinct substrate motifs recognized by different PRMTs influence how the substrate is oriented within the active site, ultimately affecting whether MMA or DMA occurs.

### 5.1. Substrate Motifs and PRMT Specificity

For Type I PRMTs, RGG/RG motifs and proline-enriched motifs are commonly found in their substrates. The RGG/RG motif is particularly enriched in proteins methylated by PRMT1, PRMT3, PRMT6, and PRMT8, with PRMT6 displaying a strong preference for RG motifs [44,62,63,64]. Unlike other Type I PRMTs, PRMT2 and PRMT4 exhibit a distinct preference for proline-enriched motifs, likely due to PRMT2’s Src homology 3 (SH3) domains, which facilitates interactions with proline-rich sequences [43,65]. For Type II PRMTs, PRMT5 preferentially methylates substrates containing GAR (glycine-arginine) motifs [43]. However, PRMT9 exhibits low substrate motif conservation, with a recognized sequence that includes K/R/F at the −2 position, R/W at the −1 position, and M/F following the core arginine residue [66], whereas the PRMT7 prefers the RXR motif [67]. These distinct substrate preferences reflect the structural constraints that influence PRMT-substrate interactions and, consequently, methylation specificity.

### 5.2. Structural Basis of PRMT-Substrate Recognition

Structural studies of PRMT–substrate complexes reveal that the substrate conformation within the PRMT active site varies. For example, in PRMT5, the substrate adopts a β-turn conformation, whereas in PRMT7, the substrate exhibits a more extended turn [50,55]. These differences are primarily dictated by residues surrounding the methylated arginine, reinforcing the idea that PRMT-recognized motifs influence substrate orientation and methylation efficiency. Interestingly, PRMT1 preferentially methylates arginine residues located near the N-terminus rather than the C-terminus, and the amount of MMA vs. ADMA generated differs among peptide substrates [68]. This suggests that the position of the methylated arginine within the substrate sequence also contributes to methylation outcomes. These findings highlight the importance of substrate structure and motif composition in determining arginine methylation specificity. Differences in PRMT–substrate interactions, substrate folding within the active site, and residues flanking the methylation site all play crucial roles in governing PRMT activity and product formation. Further research into the molecular determinants of PRMT–substrate recognition will provide deeper insights into how different PRMTs selectively regulate arginine methylation.

## 6. Conclusions and Future Perspectives

Arginine methylation is a dynamic post-translational modification with essential roles in gene expression, RNA processing, and enzymatic activity. It influences diverse cellular processes, including protein interactions, RNA binding, and phase separation. While recent proteomic advances have expanded our understanding of methylation sites and their regulatory roles, fundamental questions remain. The mechanisms governing the transition between MMA and DMA are unclear, though PRMT substrate availability, additional PTMs, and cellular stress conditions likely play a role. Understanding how PRMTs coordinate methylation events, given their overlapping substrate specificity, remains a key challenge, as does distinguishing functionally relevant methylation from incidental modifications. Future research must integrate structural biology, proteomics, and functional genomics to elucidate the regulatory networks and physiological impact of arginine methylation. High-resolution structural studies, advanced proteomic approaches, and genome-wide CRISPR screens will be critical in mapping methylation dynamics and defining its role in development and disease.

Given PRMTs’ emerging significance as therapeutic targets in cancer, neurodegeneration, and immune disorders, developing selective inhibitors and assessing their effects on global methylation patterns will be essential for precision medicine. Many PRMT1 or PRMT5 inhibitors, like GSK3368715 or GSK3326595, show significant therapeutic effects against solid tumors and are in clinical trials [69,70]. In addition, the inhibitors of other PRMTs also have been reported [71,72]. They still need further clinical tests for efficiency and safety. This provides new insights and strategies for the development of targeted treatments. Despite significant progress, much remains unknown, emphasizing the need for multidisciplinary research to unlock the full regulatory potential of arginine methylation and its therapeutic applications.

## Figures and Tables

**Table 1:** Free-energy barriers and geometric parameters for MMA and DMA formation across different PRMTs.

Methylation Formation Type	PRMT and Mutant Sites	Angular Orientation (°) (Nη2)/(Nη1)	Spatial Distance (Å) (Nη2)/(Nη1)	Free Energy (kcal/mol) (Nη2)/(Nη1)
MMA	RnPRMT3	37.4/54.6	3.6/4.9	20.4/28.5
	HsPRMT5	Approximately equal	Approximately equal	29.4/20.4
	TbPRMT7	Approximately equal	2.9/4.43	32.7/54
DMA	RnPRMT3	11.7/27	3.2/3.7	18.4/25.4
	HsPRMT5	<30/>30	Approximately equal	20.1/31.3
	TbPRMT7	≈30/>30	3.71/3.33	44.34/40.3
ADMA	TbPRMT7 E181D	Approximately equal	3.23/3.44	36.14/37.4
SDMA	TbPRMT7 E181D/Q329A	≈30/<30	3.76/2.86	45.8/32.62

The table summarizes the angular orientation, spatial distance, and calculated free-energy barriers (in kcal/mol) for methyl group transfer to the Nη2 and Nη1 atoms of the arginine guanidino group during MMA and DMA formation. Data are shown for *Rattus norvegicus* PRMT3 (RnPRMT3), *Homo sapiens* PRMT5 (HsPRMT5), and *Trypanosoma brucei* PRMT7 (TbPRMT7), as well as two TbPRMT7 mutants (E181D and E181D/Q329A) involved in ADMA and SDMA formation, respectively. Values indicate distinct preferences and catalytic efficiencies among PRMTs and mutant variants, with geometric differences contributing to free energy variations between Nη2 and Nη1 methylation sites [56,57,58,61].

## Data Availability

No new data were created or analyzed in this study.

## Supplementary Materials

The following supporting information can be downloaded at: https://www.mdpi.com/article/10.3390/cells14110796/s1, Table S1: The summary of detected arginine methylation sites and Uniprot accession number of corresponding proteins; Tables S2 and S3: The results of GO analysis of the proteins containing MMA or DMA only; Table S4: The list of proteins whose arginine methylation levels are regulated by specific PRMTs.

## References

[1] Deribe Y.L., Pawson T., Dikic I. (2010). Post-translational modifications in signal integration. Nat. Struct. Mol. Biol..

[2] Ambler R.P., Rees M.W. (1959). Epsilon-N-Methyl-lysine in bacterial flagellar protein. Nature.

[3] Biggar K.K., Li S.S. (2015). Non-histone protein methylation as a regulator of cellular signalling and function. Nat. Rev. Mol. Cell Biol..

[4] Fulton M.D., Brown T., Zheng Y.G. (2019). The Biological Axis of Protein Arginine Methylation and Asymmetric Dimethylarginine. Int. J. Mol. Sci..

[5] Guccione E., Richard S. (2019). The regulation, functions and clinical relevance of arginine methylation. Nat. Rev. Mol. Cell Biol..

[6] Wu Q., Schapira M., Arrowsmith C.H., Barsyte-Lovejoy D. (2021). Protein arginine methylation: From enigmatic functions to therapeutic targeting. Nat. Rev. Drug Discov..

[7] Xu J., Richard S. (2021). Cellular pathways influenced by protein arginine methylation: Implications for cancer. Mol. Cell.

[8] Fuhrmann J., Clancy K.W., Thompson P.R. (2015). Chemical biology of protein arginine modifications in epigenetic regulation. Chem. Rev..

[9] Fischer N., Neumann P., Konevega A.L., Bock L.V., Ficner R., Rodnina M.V., Stark H. (2015). Structure of the E. coli ribosome-EF-Tu complex at <3 A resolution by Cs-corrected cryo-EM. Nature.

[10] Pellegrino S., Dent K.C., Spikes T., Warren A.J. (2023). Cryo-EM reconstruction of the human 40S ribosomal subunit at 2.15 A resolution. Nucleic Acids Res..

[11] Yip K.M., Fischer N., Paknia E., Chari A., Stark H. (2020). Atomic-resolution protein structure determination by cryo-EM. Nature.

[12] Bremang M., Cuomo A., Agresta A.M., Stugiewicz M., Spadotto V., Bonaldi T. (2013). Mass spectrometry-based identification and characterisation of lysine and arginine methylation in the human proteome. Mol. Biosyst..

[13] Geoghegan V., Guo A., Trudgian D., Thomas B., Acuto O. (2015). Comprehensive identification of arginine methylation in primary T cells reveals regulatory roles in cell signalling. Nat. Commun..

[14] Guo A., Gu H., Zhou J., Mulhern D., Wang Y., Lee K.A., Yang V., Aguiar M., Kornhauser J., Jia X. (2014). Immunoaffinity enrichment and mass spectrometry analysis of protein methylation. Mol. Cell. Proteom..

[15] Sylvestersen K.B., Horn H., Jungmichel S., Jensen L.J., Nielsen M.L. (2014). Proteomic analysis of arginine methylation sites in human cells reveals dynamic regulation during transcriptional arrest. Mol. Cell. Proteom..

[16] Ong S.E., Mittler G., Mann M. (2004). Identifying and quantifying in vivo methylation sites by heavy methyl SILAC. Nat. Methods.

[17] Uhlmann T., Geoghegan V.L., Thomas B., Ridlova G., Trudgian D.C., Acuto O. (2012). A method for large-scale identification of protein arginine methylation. Mol. Cell. Proteom..

[18] Larsen S.C., Sylvestersen K.B., Mund A., Lyon D., Mullari M., Madsen M.V., Daniel J.A., Jensen L.J., Nielsen M.L. (2016). Proteome-wide analysis of arginine monomethylation reveals widespread occurrence in human cells. Sci. Signal..

[19] Li Z., Wang Q., Wang Y., Wang K., Liu Z., Zhang W., Ye M. (2021). An efficient approach based on basic strong cation exchange chromatography for enriching methylated peptides with high specificity for methylproteomics analysis. Anal. Chim. Acta.

[20] Ma M., Zhao X., Chen S., Zhao Y., Yang L., Feng Y., Qin W., Li L., Jia C. (2017). Strategy Based on Deglycosylation, Multiprotease, and Hydrophilic Interaction Chromatography for Large-Scale Profiling of Protein Methylation. Anal. Chem..

[21] Wang K., Dong M., Mao J., Wang Y., Jin Y., Ye M., Zou H. (2016). Antibody-Free Approach for the Global Analysis of Protein Methylation. Anal. Chem..

[22] Hartel N.G., Chew B., Qin J., Xu J., Graham N.A. (2019). Deep Protein Methylation Profiling by Combined Chemical and Immunoaffinity Approaches Reveals Novel PRMT1 Targets. Mol. Cell. Proteom..

[23] Lu L., Ye Z., Zhang R., Olsen J.V., Yuan Y., Mao Y. (2024). ETD-Based Proteomic Profiling Improves Arginine Methylation Identification and Reveals Novel PRMT5 Substrates. J. Proteome Res..

[24] Wang Q., Li Z., Zhou J., Wang Y., Wang K., Qin H., Ye M. (2021). Chemical Depletion of Histidine-Containing Peptides Allows Identification of More Low-Abundance Methylation Sites from Proteome Samples. J. Proteome Res..

[25] Wang Q., Li Z., Zhang S., Li Y., Wang Y., Fang Z., Ma Y., Liu Z., Zhang W., Li D. (2022). Global profiling of arginine dimethylation in regulating protein phase separation by a steric effect-based chemical-enrichment method. Proc. Natl. Acad. Sci. USA.

[26] Liu Z., Fang Z., Wang K., Ye M. (2023). Hydrophobic Derivatization Strategy Facilitates Comprehensive Profiling of Protein Methylation. J. Proteome Res..

[27] Wang Q., Yan X., Fu B., Xu Y., Li L., Chang C., Jia C. (2023). mNeuCode Empowers Targeted Proteome Analysis of Arginine Dimethylation. Anal. Chem..

[28] Wang J., Wang Q., Zhou J., Wang Y., Liu Z., Wang K., Ye M. (2024). A Chemoenzymatic Method Enables Global Enrichment and Characterization of Protein Arginine Methylation. Anal. Chem..

[29] Bedford M.T., Richard S. (2005). Arginine methylation an emerging regulator of protein function. Mol. Cell.

[30] Gui S., Wooderchak-Donahue W.L., Zang T., Chen D., Daly M.P., Zhou Z.S., Hevel J.M. (2013). Substrate-induced control of product formation by protein arginine methyltransferase 1. Biochemistry.

[31] Evich M., Stroeva E., Zheng Y.G., Germann M.W. (2016). Effect of methylation on the side-chain pKa value of arginine. Protein Sci..

[32] Santos M. (2024). Conformational analysis of arginine methylated and di methylated and serine phosphorylated in silico. Preprint.

[33] Ryan V.H., Dignon G.L., Zerze G.H., Chabata C.V., Silva R., Conicella A.E., Amaya J., Burke K.A., Mittal J., Fawzi N.L. (2018). Mechanistic View of hnRNPA2 Low-Complexity Domain Structure, Interactions, and Phase Separation Altered by Mutation and Arginine Methylation. Mol. Cell.

[34] Wang Y.P., Zhou W., Wang J., Huang X., Zuo Y., Wang T.S., Gao X., Xu Y.Y., Zou S.W., Liu Y.B. (2016). Arginine Methylation of MDH1 by CARM1 Inhibits Glutamine Metabolism and Suppresses Pancreatic Cancer. Mol. Cell.

[35] Wang K., Luo L., Fu S., Wang M., Wang Z., Dong L., Wu X., Dai L., Peng Y., Shen G. (2023). PHGDH arginine methylation by PRMT1 promotes serine synthesis and represents a therapeutic vulnerability in hepatocellular carcinoma. Nat. Commun..

[36] Lei Y., Han P., Chen Y., Wang H., Wang S., Wang M., Liu J., Yan W., Tian D., Liu M. (2022). Protein arginine methyltransferase 3 promotes glycolysis and hepatocellular carcinoma growth by enhancing arginine methylation of lactate dehydrogenase A. Clin. Transl. Med..

[37] Lim Y., Lee J.Y., Ha S.J., Yu S., Shin J.K., Kim H.C. (2020). Proteome-wide identification of arginine methylation in colorectal cancer tissues from patients. Proteome Sci..

[38] Ma M., Liu F., Miles H.N., Kim E.J., Fields L., Xu W., Li L. (2023). Proteome-wide Profiling of Asymmetric Dimethylated Arginine in Human Breast Tumors. J. Am. Soc. Mass Spectrom..

[39] Onwuli D.O., Rigau-Roca L., Cawthorne C., Beltran-Alvarez P. (2017). Mapping arginine methylation in the human body and cardiac disease. Proteom. Clin. Appl..

[40] Yang C.Y., Chiu L.L., Chang C.C., Chuang H.C., Tan T.H. (2018). Induction of DUSP14 ubiquitination by PRMT5-mediated arginine methylation. FASEB J..

[41] Aikio M., Odeh H.M., Wobst H.J., Lee B.L., Chan U., Mauna J.C., Mack K.L., Class B., Ollerhead T.A., Ford A.F. (2025). Opposing roles of p38alpha-mediated phosphorylation and PRMT1-mediated arginine methylation in driving TDP-43 proteinopathy. Cell Rep..

[42] Tang J., Frankel A., Cook R.J., Kim S., Paik W.K., Williams K.R., Clarke S., Herschman H.R. (2000). PRMT1 is the predominant type I protein arginine methyltransferase in mammalian cells. J. Biol. Chem..

[43] Li W.J., He Y.H., Yang J.J., Hu G.S., Lin Y.A., Ran T., Peng B.L., Xie B.L., Huang M.F., Gao X. (2021). Profiling PRMT methylome reveals roles of hnRNPA1 arginine methylation in RNA splicing and cell growth. Nat. Commun..

[44] Hamey J.J., Rakow S., Bouchard C., Senst J.M., Kolb P., Bauer U.M., Wilkins M.R., Hart-Smith G. (2021). Systematic investigation of PRMT6 substrate recognition reveals broad specificity with a preference for an RG motif or basic and bulky residues. FEBS J..

[45] Musiani D., Bok J., Massignani E., Wu L., Tabaglio T., Ippolito M.R., Cuomo A., Ozbek U., Zorgati H., Ghoshdastider U. (2019). Proteomics profiling of arginine methylation defines PRMT5 substrate specificity. Sci. Signal..

[46] Radzisheuskaya A., Shliaha P.V., Grinev V., Lorenzini E., Kovalchuk S., Shlyueva D., Gorshkov V., Hendrickson R.C., Jensen O.N., Helin K. (2019). PRMT5 methylome profiling uncovers a direct link to splicing regulation in acute myeloid leukemia. Nat. Struct. Mol. Biol..

[47] Zhang X., Zhou L., Cheng X. (2000). Crystal structure of the conserved core of protein arginine methyltransferase PRMT3. EMBO J..

[48] Tewary S.K., Zheng Y.G., Ho M.-C. (2019). Protein arginine methyltransferases: Insights into the enzyme structure and mechanism at the atomic level. Cell. Mol. Life Sci..

[49] Zhang X., Cheng X. (2003). Structure of the predominant protein arginine methyltransferase PRMT1 and analysis of its binding to substrate peptides. Structure.

[50] Antonysamy S., Bonday Z., Campbell R.M., Doyle B., Druzina Z., Gheyi T., Han B., Jungheim L.N., Qian Y., Rauch C. (2012). Crystal structure of the human PRMT5:MEP50 complex. Proc. Natl. Acad. Sci. USA.

[51] Cura V., Troffer-Charlier N., Wurtz J.M., Bonnefond L., Cavarelli J. (2014). Structural insight into arginine methylation by the mouse protein arginine methyltransferase 7: A zinc finger freezes the mimic of the dimeric state into a single active site. Acta Crystallogr. D Biol. Crystallogr..

[52] Hasegawa M., Toma-Fukai S., Kim J.D., Fukamizu A., Shimizu T. (2014). Protein arginine methyltransferase 7 has a novel homodimer-like structure formed by tandem repeats. FEBS Lett..

[53] Rossi V., Nielson S.E., Ortolano A., Lonardo I., Haroldsen E., Comer D., Price O.M., Wallace N., Hevel J.M. (2024). Oligomerization of protein arginine methyltransferase 1 and its effect on methyltransferase activity and substrate specificity. Protein Sci..

[54] Price O.M., Thakur A., Ortolano A., Towne A., Velez C., Acevedo O., Hevel J.M. (2021). Naturally occurring cancer-associated mutations disrupt oligomerization and activity of protein arginine methyltransferase 1 (PRMT1). J. Biol. Chem..

[55] Wang C., Zhu Y., Caceres Tamar B., Liu L., Peng J., Wang J., Chen J., Chen X., Zhang Z., Zuo X. (2014). Structural Determinants for the Strict Monomethylation Activity by *Trypanosoma brucei* Protein Arginine Methyltransferase 7. Structure.

[56] Chu Y., Li G., Guo H. (2013). QM/MM MD and free energy simulations of the methylation reactions catalyzed by protein arginine methyltransferase PRMT3. Can. J. Chem..

[57] Yue Y., Chu Y., Guo H. (2015). Computational Study of Symmetric Methylation on Histone Arginine Catalyzed by Protein Arginine Methyltransferase PRMT5 through QM/MM MD and Free Energy Simulations. Molecules.

[58] Ren W.S., Jiang K.B., Deng H., Lu N., Yu T., Guo H., Qian P. (2020). Catalytic Mechanism and Product Specificity of Protein Arginine Methyltransferase PRMT7: A Study from QM/MM Molecular Dynamics and Free Energy Simulations. J. Chem. Theory Comput..

[59] Debler E.W., Jain K., Warmack R.A., Feng Y., Clarke S.G., Blobel G., Stavropoulos P. (2016). A glutamate/aspartate switch controls product specificity in a protein arginine methyltransferase. Proc. Natl. Acad. Sci. USA.

[60] Jain K., Warmack R.A., Debler E.W., Hadjikyriacou A., Stavropoulos P., Clarke S.G. (2016). Protein Arginine Methyltransferase Product Specificity Is Mediated by Distinct Active-site Architectures. J. Biol. Chem..

[61] Ren W.S., Rahman A., Jiang K.B., Deng H., Zhao Y.Y., Zhang W.J., Liu K., Qian P., Guo H. (2022). Unraveling the Origins of Changing Product Specificity Properties of Arginine Methyltransferase PRMT7 by the E181D and E181D/Q329A Mutations through QM/MM MD and Free-Energy Simulations. J. Chem. Theory Comput..

[62] Hamey J.J., Separovich R.J., Wilkins M.R. (2018). MT-MAMS: Protein Methyltransferase Motif Analysis by Mass Spectrometry. J. Proteome Res..

[63] Pahlich S., Zakaryan R.P., Gehring H. (2008). Identification of proteins interacting with protein arginine methyltransferase 8: The Ewing sarcoma (EWS) protein binds independent of its methylation state. Proteins.

[64] Zhu J., Li X., Cai X., Zhou Z., Liao Q., Liu X., Wang J., Xiao W. (2023). Asymmetric arginine dimethylation of cytosolic RNA and DNA sensors by PRMT3 attenuates antiviral innate immunity. Proc. Natl. Acad. Sci. USA.

[65] Ren R., Mayer B.J., Cicchetti P., Baltimore D. (1993). Identification of a ten-amino acid proline-rich SH3 binding site. Science.

[66] Gayatri S., Cowles M.W., Vemulapalli V., Cheng D., Sun Z.W., Bedford M.T. (2016). Using oriented peptide array libraries to evaluate methylarginine-specific antibodies and arginine methyltransferase substrate motifs. Sci. Rep..

[67] Feng Y., Maity R., Whitelegge J.P., Hadjikyriacou A., Li Z., Zurita-Lopez C., Al-Hadid Q., Clark A.T., Bedford M.T., Masson J.Y. (2013). Mammalian protein arginine methyltransferase 7 (PRMT7) specifically targets RXR sites in lysine- and arginine-rich regions. J. Biol. Chem..

[68] Osborne T.C., Obianyo O., Zhang X., Cheng X., Thompson P.R. (2007). Protein arginine methyltransferase 1: Positively charged residues in substrate peptides distal to the site of methylation are important for substrate binding and catalysis. Biochemistry.

[69] Feustel K., Falchook G.S. (2022). Protein Arginine Methyltransferase 5 (PRMT5) Inhibitors in Oncology Clinical Trials: A review. J. Immunother. Precis. Oncol..

[70] Wu J., Li D., Wang L. (2024). Overview of PRMT1 modulators: Inhibitors and degraders. Eur. J. Med. Chem..

[71] Cao M., Nguyen T., Song J., Zheng Y.G. (2025). Biomedical effects of protein arginine methyltransferase inhibitors. J. Biol. Chem..

[72] Dong H., He X., Zhang L., Chen W., Lin Y.C., Liu S.B., Wang H., Nguyen L.X.T., Li M., Zhu Y. (2024). Targeting PRMT9-mediated arginine methylation suppresses cancer stem cell maintenance and elicits cGAS-mediated anticancer immunity. Nat. Cancer.

